# Evaluation of Color Stability and Marginal Integrity in Provisional Restorations: A Study of Milling, 3D Printing, and Conventional Fabrication Methods

**DOI:** 10.3390/dj13050189

**Published:** 2025-04-25

**Authors:** Austin Galbraith, Mai Doan, Tyson Galbraith, Neamat Hassan Abubakr

**Affiliations:** 1 School of Dental Medicine, University of Nevada, Las Vegas, NV 89557, USA; galbra4@unlv.nevada.edu (A.G.); doanm1@unlv.nevada.edu (M.D.); galbrt1@unlv.nevada.edu (T.G.); 2 Department of Biomedical Sciences, School of Dental Medicine, University of Nevada, Las Vegas, NV 89557, USA

**Keywords:** provisional restorations, temporary restorations, CAD/CAM, 3D printing, acrylic, bis-acryl

## Abstract

**Background:** The quality of a provisional restoration, especially its color and marginal integrity, can play a critical role in its survival and overall patient satisfaction. This study aims to evaluate the color stability and marginal fit differences between provisional restorations fabricated by non-traditional methods compared to manual fabrication. **Methods:** A total of 80 extracted teeth were prepared for ceramic crowns and randomly divided into four groups: acrylic, 3D printing, computer-aided design/computer-aided manufacturing (CAD/CAM), and bis-acryl. The examined teeth were subjected to artificial aging using a thermocycling machine dwelling for 5000 cycles (simulating 6 clinical months). Color stability and marginal integrity were measured before and after thermal aging using a VITA Easyshade V spectrophotometer and 3D surface non-contact profilometer. ANOVA was used to determine whether the mean value difference was significantly different. **Results:** The 3D-printed and bis-acryl provisional crowns displayed the lowest change in marginal integrity, while the acrylic provisional crowns showed the greatest change in marginal integrity (*p* = 0.0001). Additionally, the acrylic provisional material revealed a significantly greater color change. **Conclusions:** The 3D-printed provisional crowns demonstrated the best marginal integrity and color stability.

## 1. Introduction

A critical factor in successful treatment in fixed prosthodontics is the quality of provisional restorations. A successful provisional restoration must fulfill the functional and esthetic requirements until the patient receives the definitive restoration [1]. An important indicator of a clinically acceptable crown is the marginal integrity, as a larger gap between the tooth and crown can lead to biofilm formation [2]. Among several factors of patient dissatisfaction, especially in the esthetic zones, is the discoloration of provisional restorations [3]. Restorations should offer an adequate fit to ensure the mechanical stability and durability of the restoration, which are essential and serve many functions including protection of the pulpal tissues, prevention of bacterial contamination, and preservation of periodontal tissues [4,5]. Provisional restorations protect prepared teeth and maintain esthetics, function, and periodontal health while a patient awaits the placement of a definitive crown or bridge. Two qualities that are particularly important in these temporary restorations are color stability and marginal fit. Specifically, poor color stability can cause esthetic dissatisfaction or self-consciousness, while an inadequate marginal fit can result in secondary caries or gingival irritation and compromise the final restoration’s success.

Ensuring that a provisional restoration holds up well both esthetically (color) and biologically (marginal adaptation) is therefore paramount to maintaining patient satisfaction and oral health until the final restoration is placed. While numerous provisional materials are available, direct comparisons of their color stability and marginal adaptation over a clinically relevant timeframe are limited.

Techniques and materials used to fabricate provisional restorations vary greatly in their physical and mechanical properties such as color stability, dimensional stability, and polishability [6,7]. Recently, computer-aided design/computer-aided manufacturing (CAD/CAM) and 3D printing technologies have been utilized to fabricate provisional restorations, providing various options for fabricating provisional prostheses [8,9,10].

Since the advent of CAD/CAM as part of the “digital revolution” in dentistry, various digital scanners and milling machines have been introduced. The rapid acceptance of CAD/CAM took place in part due to the speed and quality of fabricating materials [11,12]. Some say a “second revolution” will take place when layered fabrication techniques, or 3D printing, can accurately produce similar results to traditional techniques [12]. This may already be the case because when examining the marginal fit and internal adaptation of 3D provisional crowns compared to CAD/CAM-milled provisionals, the 3D-printed provisional crowns proved to be superior [13]. CAD/CAM-milled crowns have better physical properties, but 3D-printed crowns have better mechanical properties [14].

The use of non-traditional methods (milled and 3D-printed) of fabricating temporaries is of interest as technology improves, as it allows virtual wax-ups, enabling quicker and more predictable fabrication [15]. Three-dimensional printing is gaining popularity in dentistry due to its low-cost manufacturing compared to CAD/CAM prostheses, and commercially available 3D-printable light-polymerizing resin materials have sufficient mechanical properties for provisional restorations [16].

For traditional provisional restorations, acrylic resin has been used in the past due to many properties, especially its high strength and physical requirements [17]. Despite these advantages, some disadvantages include heat production, high polymerization shrinkage when setting, and poor staining resistance. These shortcomings are mainly overcome with bis-acryl resin [18,19]. When evaluating the contour, marginal integrity, occlusion, and finish of bis-acryl provisionals, polymethyl methacrylate (PMMA) was found to be deficient in the majority of categories, with the surface finish being the exception [20].

Two factors, however, namely marginal integrity and color stability, are of utmost importance to the success of a provisional. A well-adapted margin on a provisional restoration has a decreased amount of plaque and caries, and an increase in cement effectiveness [21,22]. There is a well-established relationship between the accuracy of margin proximity of the tooth and the integrity of the periodontium [23]. It is vital that tooth and gingival health are maintained during this prolonged stage of treatment so that finals can be delivered to a healthy mouth ready to receive them. It is no surprise that this factor is important to examine, especially as it pertains to aging. Marginal breakdown is bound to occur with time, so measuring this precisely can give clinicians a good guide for how long a provisional may be expected to last without negative consequences. The other key factor would be the color stability of the provisional, which is essential for esthetic areas. If a color change occurs, the incorrect shade may be selected for the final restoration. Additionally, it may affect the acceptability of the temporary while in use, leading to patient dissatisfaction [24]. The null hypothesis is that the fabrication techniques used for provisional restorations do not affect their color stability and marginal integrity. This study aims to investigate the marginal integrity and color stability of provisional restorations fabricated by four different techniques (bis-acryl, acrylic, CAD/CAM-milled, and 3D-printed) after thermal aging.

## 2. Materials and Methods

**Figure 1:**
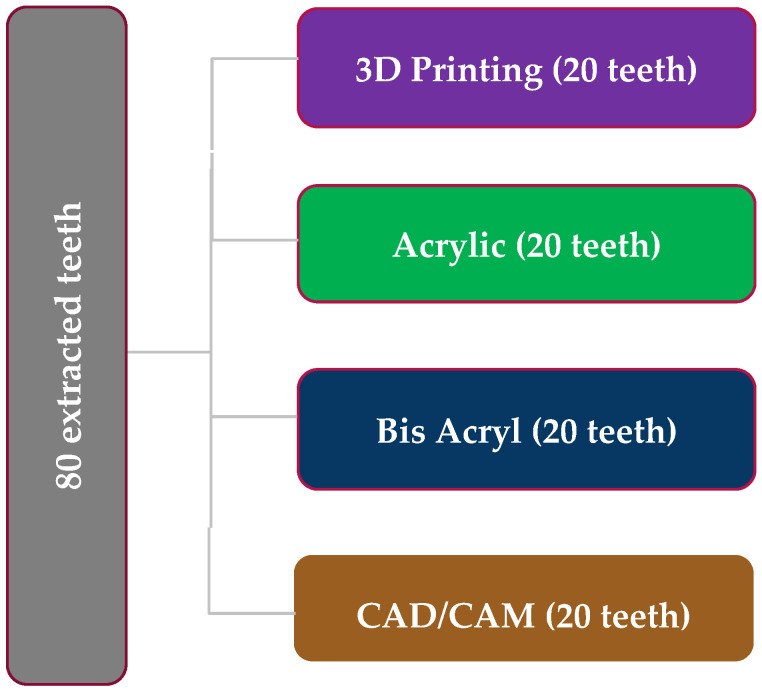
Descriptive flowchart for all examined materials.

The calculated sample size was 60 teeth, and the effect size was calculated based on an alpha error of 0.05 and a beta of 0.2, with a required power of 80%. This was based on the results of a reference article [4]. The sample size was increased to 80 to account for any failure/loss/fracture of the samples during the experiments. A total of 80 extracted anterior teeth were obtained from the Oral Surgery Clinic at UNLV. Teeth exhibiting significant imperfections were excluded from the study, including those with visible fractures, extensive composite restorations, composites extending to the anticipated crown margin, or any form of carious lesion (Figure 1).

**Figure 2:**
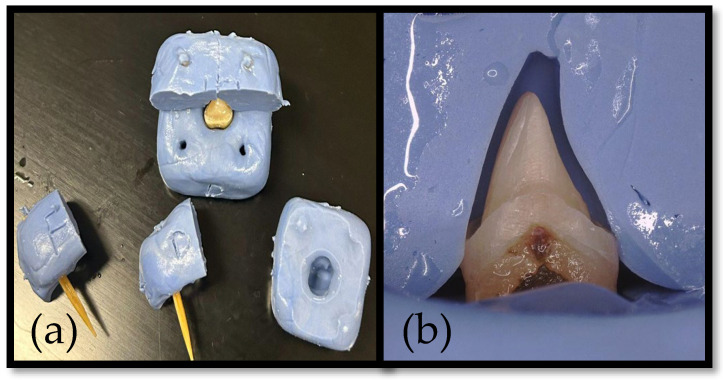
(**a**) PVS matrix used as preparation guide. (**b**) Cross-sections of PVS matrix.

The selected teeth were sterilized and embedded in a putty base. A putty matrix was utilized to obtain impressions and fabricate a temporary crown. To ensure adequate tooth reduction, a second putty matrix was fabricated and sectioned in a manner similar to the first (Figure 2a,b).

Once the putty matrices were completed, the teeth were prepared for ceramic crowns. The specimens were randomly assigned to one of four groups (bis-acryl, acrylic, milled, or 3D-printed) to evaluate marginal adaptation and color stability over a simulated six-month period.

Thermal aging was performed using a thermocycler (SD Mechatronik, Westerham, Germany) with water baths maintained at 5 °C and 55 °C, with an immersion duration of 20 s per cycle. Measurements were recorded at 5000 thermocycles, which is equivalent to approximately half a clinical year [25]. Each group consisted of 20 teeth.

The teeth were digitally scanned for the 3D printing and CAD/CAM groups, and the generated designs were utilized for milling CAD/CAM restorations and fabricating 3D-printed crowns. The temporary crown fabrication methods evaluated in this study included the following groups: acrylic material (SNAP^®^ Self-Cure Resin, Parkell, Edgewood, NY, USA), 3D printing—methacrylate-based resin (VeriModel™ OS, WhipMix, Louisville, KY, USA), CAD/CAM-polymethyl methacrylate (PMMA) (Telio, Ivoclar Vivadent, Ellwangen, Germany), and bis-acryl (MaxiTemp HP, Henry Schein, Melville, NY, USA) (Table 1).

Using the previously created putty matrices, the acrylic SNAP^®^ material (Parkell) was mixed and poured into the matrices. The prepared tooth was then inserted into the putty for shaping. Once set, the fabricated crown was polished using Shofu discs and OneGloss points. (SHOFU Dental Corporation, San Marcos, CA, USA). A similar process was followed for the bis-acryl material, with Vaseline applied to the teeth to facilitate the removal of the temporary crown from the tooth. The bis-acryl crowns were polished in the same manner as the acrylic crowns.

For the CAD/CAM group (Telio CAD/CAM crowns), each of the 20 teeth was digitally scanned using a Planmeca Emerald scanner. (Planmeca USA Inc., Charlotte, NC, USA). crown design was digitally created and milled using a Planmeca 40S milling machine (Planmeca USA Inc., Charlotte, NC, USA).

The 3D-printed group was fabricated using an Asiga 3D printer using VeriModel™ OS, 3D print Resin. Upon completion, the 3D-printed temporary crowns were separated from the base and underwent ultrasonic cleaning in 98% pure isopropyl alcohol for 10 min. The isopropyl alcohol was then replaced, and the crowns underwent an additional 5 min cleaning cycle. As a final step, each temporary crown was rinsed with water before use.

Each of the 80 temporary crowns was cemented onto the prepared teeth using RelyX™ Temporary, a zinc oxide-based temporary cement (3M RelyX Temp NE, 3M, USA).

**Figure 3:**
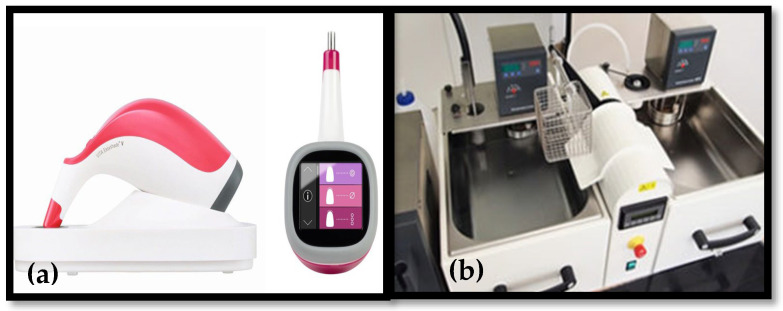
(**a**) VITA Easyshade V (VITA North America, Brea, CA, USA); (**b**) thermal aging machine.

Marginal adaptation was assessed at a specific measurement point on each tooth using a 3D surface profilometer at 40× magnification (VR-3100; Keyence, Keyence Corporation, Osaka, Japan). The recorded reading was the average of six readings. Additionally, shade measurements were recorded before and after the study using a VITA Easyshade V spectrophotometer (VITA Zahnfabrik H. Rauter GmbH & Co. KG, Bad Säckingen, Germany) (Figure 3a). ΔE was calculated using the following formula:

$$\Delta E_{ab}^{*}=\sqrt{{\left(L_{2}^{*}-L_{1}^{*}\right)}^{2}+{\left(a_{2}^{*}-a_{1}^{*}\right)}^{2}+{\left(b_{2}^{*}-b_{1}^{*}\right)}^{2}}$$

To simulate six months of intraoral aging, a thermocycler (Figure 3b) was used 24 h after fabricating the provisional restorations, subjecting the specimens to 5000 thermal cycles between 5 °C and 55 °C. Following thermocycling, margins were reassessed, and shade measurements were repeated to evaluate changes in color stability.

Statistical Analysis: SPSS 28 (SPSS, IBM, Armonk, NY, USA) was used for statistical calculations. Analysis of the data was performed using analysis of variance (ANOVA) to detect changes between the groups and within the groups.

## 3. Results

A statistically significant difference in marginal integrity was observed among the milled CAD/CAM, acrylic, bis-acryl, and 3D-printed provisional crown groups (*p* = 1.1 × 10^−5^ < 0.05). After six simulated clinical months, the results demonstrated a significant variation in marginal adaptation between the groups. Due to the brittle nature of extracted teeth, marginal breakdown occurred in 1 anterior tooth, leaving 19 specimens in the 3D-printed group (Table 2). Based on the ANOVA test (Table 3), the findings confirmed a statistically significant difference in marginal integrity among the milled CAD/CAM, acrylic, bis-acryl, and 3D-printed provisional groups (*p* = 1.1 × 10^−5^ < 0.05). The average marginal gap for each group was as follows:

⭘ 3D-printed: 0.116 ± 0.009 µm;
⭘ Acrylic: 0.30 ± 0.03 µm;
⭘ Bis-acryl: 0.117 ± 0.007 µm;
⭘ CAD/CAM: 0.18 ± 0.01 µm.

Among the tested materials, the 3D-printed and bis-acryl provisional crowns exhibited the lowest change in marginal integrity, with average values of 0.1167 mm and 0.1175 mm, respectively. In contrast, acrylic provisional crowns displayed the most significant change in marginal integrity, with an average of 0.30 mm.

**Figure 4:**
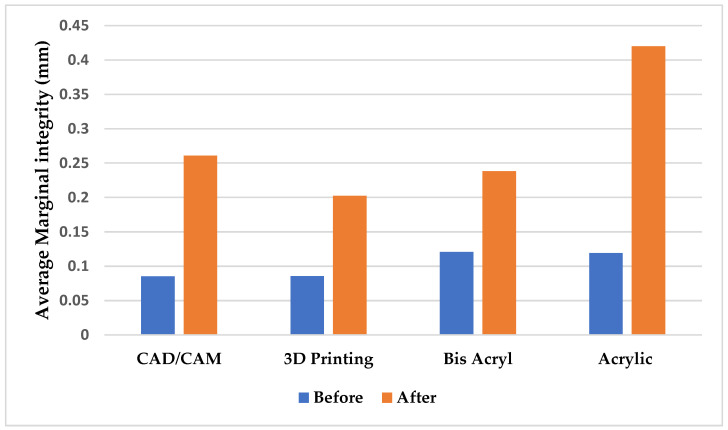
Average marginal integrity of all examined provisional crowns after six clinical months.

The one-way ANOVA test confirmed that the *p*-value was less than 0.05 for the variable marginal gap, leading to the rejection of the null hypothesis. Thus, a statistically significant difference was present among the tested materials, indicating differences in marginal integrity between the milled CAD/CAM, acrylic, bis-acryl, and 3D-printed provisional crowns. The milled CAD/CAM group exhibited moderate marginal integrity, with an average marginal gap of 0.176 mm. After thermal aging, the results indicated a statistically significant difference in marginal integrity among the groups (*p* = 0.00001) (Figure 4).

Color stability was evaluated using an Easyshade V. The conventional acrylic group exhibited the greatest overall color shift, with a mean ΔE* of 3.98 ± 0.47. This was driven by a marked decrease in lightness (ΔL* −2.31 ± 0.35) and shifts toward red and yellow (Δa* +1.13 ± 0.21; Δb* +2.41 ± 0.44). The bis-acryl group showed moderate discoloration (mean ΔE* 1.72 ± 0.32), accompanied by smaller changes in lightness (ΔL* −0.98 ± 0.18), red–green axis (Δa* +0.64 ± 0.13), and yellow–blue axis (Δb* +1.27 ± 0.29). The milled PMMA group demonstrated minimal color alteration (mean ΔE* 0.89 ± 0.18), with only slight decreases in lightness (ΔL* −0.42 ± 0.09) and modest positive shifts in Δa* (+0.21 ± 0.08) and Δb* (+0.69 ± 0.15). Meanwhile, the 3D-printed group remained essentially unchanged, recording a mean ΔE* of 0.46 ± 0.11 (ΔL* −0.18 ± 0.07; Δa* +0.12 ± 0.05; Δb* +0.31 ± 0.09). These comprehensive CIELAB data confirm that thermocycling-induced discoloration is the highest in conventional acrylic, intermediate in bis-acryl, and minimal in both milled and 3D-printed materials, thereby underscoring the superior color stability of CAD/CAM-fabricated provisionals (Table 4).

## 4. Discussion

Provisional restorations must possess optimum color stability, marginal integrity, and surface characteristics. In the present investigation, four commercially available provisional systems were examined, each with a distinct resin chemistry and corresponding milling or 3D printing protocols to mirror the broad spectrum of techniques currently used in clinical practice. This “real-world” approach allows clinicians to see how each market-leading material performs under its intended workflow. The null hypothesis was rejected; within the constraints of the present study, the 3D-printed group outperformed all others in both marginal integrity and color stability. Manually fabricated bis-acryl crowns exhibited superior marginal adaptation compared to their CAD/CAM counterparts, whereas the acrylic group demonstrated the poorest performance across both parameters.

A provisional restoration with compromised marginal integrity may permit the ingress of saliva and oral pathogens, leading to complications such as dental sensitivity and pulp inflammation which will lead to the discomfort of patients. Consequently, achieving a provisional restoration with proper marginal adaptation is essential to safeguard both the functional and biological integrity of the tooth while providing patients with satisfactory esthetics and comfort. A commonly accepted guideline places the clinically acceptable marginal gap at or below 120 µm, although some studies suggest ideal margins may be under 100 µm [26].

The findings of this study demonstrate a statistically significant difference in marginal integrity among milled CAD/CAM, acrylic, bis-acryl, and 3D-printed provisional crowns (*p* = 1.1 × 10^−5^ < 0.05), with 3D-printed and bis-acryl materials exhibiting the lowest change in marginal adaptation over six simulated clinical months. These findings align with previous research suggesting that 3D-printed provisionals provide superior marginal fit compared to CAD/CAM and conventional acrylic-based materials [14,27]. Marginal adaptation is a critical determinant of the success and longevity of provisional restorations, as poor adaptation can lead to microleakage, plaque accumulation, secondary caries, and periodontal complications. In this study, the 3D-printed group exhibited the lowest marginal discrepancies, highlighting the precision and consistency achievable through additive manufacturing. Unlike manual fabrication methods, 3D printing relies on a fully digital workflow. It builds the restoration in a layer-by-layer manner, reducing cumulative errors and ensuring a more accurate reproduction of the intended design. Moreover, 3D printing resins are formulated to optimize mechanical and esthetic properties, helping to maintain marginal adaptation and resist discoloration. These factors—enhanced precision through digital design, controlled layer fabrication, and advanced material compositions—likely contributed to the superior performance observed for 3D-printed restorations in this study. As the technology evolves, 3D printing is becoming an increasingly viable alternative for clinicians seeking improved accuracy and predictable outcomes in provisional restoration fabrication. Previous research has shown that subtractive manufacturing techniques allow for enhanced precision and better internal and marginal adaptation compared to conventional acrylic materials [28]. The milled CAD/CAM group exhibited moderate marginal integrity (0.176 mm), suggesting that although CAD/CAM milling technology produces better adaptation than acrylic, it may still be inferior to 3D-printed and bis-acryl materials. These findings further support the conclusion that acrylic provisionals may not be ideal for long-term use due to their tendency to undergo deformation and increased marginal gap formation over time.

In the present investigation, marginal integrity significantly decreased following the aging treatment, suggesting that all fabrication techniques experienced some degree of degradation after six months of thermal aging, simulating oral service conditions. Several studies indicated that thermal aging or thermal stress affects the marginal integrity of restorative materials [29,30]. The findings of this study indicate that both the type of provisional restorative material and the simulated aging process had a significant impact on the marginal gap of provisional crowns. Additionally, the interaction between these two factors further influenced the extent of marginal adaptation, emphasizing the importance of material selection and clinical conditions in maintaining restoration integrity over time.

In addition to marginal adaptation, color stability is another essential parameter for the clinical success of provisional restorations. The present study revealed that acrylic provisional crowns exhibited the most significant color change following thermocycling, with a 30% discoloration. In contrast, the milled, bis-acryl, and 3D-printed groups demonstrated significantly less discoloration. These findings are consistent with prior research indicating that acrylic-based materials are more susceptible to staining and color degradation [31,32]. In 2020, Song et al. reported no statistical difference in the color stability of CAD/CAM and 3D-printed provisional restorations [33]. More significant increases in the discoloration and surface roughness of provisional restorations are linked to the length of time they remain intraorally. This is influenced by diet, oral hygiene, chemical reactivity, and water sorption. However, it is important to note that effective polishing protocols play a crucial role in plaque prevention, especially when the restorations are in service for a prolonged period [34,35].

The resin matrix formulation (e.g., polymethyl methacrylate versus bis-acryl composites), degree of conversion, and cross-link density critically determine water sorption and chromogen uptake [36]. Milled PMMA blocks typically exhibit very low residual monomer content and a highly cross-linked polymer network, conferring greater hydrophobicity and resistance to staining. Conventional auto-polymerized PMMA, by contrast, often retains more unreacted monomer and has a less dense network, leading to increased water uptake and pigment penetration. Photopolymerized 3D-printed resins—comprising various methacrylate monomers and photoinitiators—can differ in filler type, particle size distribution, and degree of polymerization; lower conversion and micro-porosities in these materials tend to promote greater sorption of dietary chromogens [37]. Furthermore, the chemical stability of incorporated pigments and the polarity of the polymer matrix modulate the affinity for staining agents: highly polar matrices attract hydrophilic chromogens (e.g., coffee and tea), whereas more hydrophobic networks better resist pigment adsorption [38]. These compositional factors together explain why, in our study, milled PMMA showed superior color stability compared to both conventional and 3D-printed materials. Clinicians should, therefore, consider the fabrication method and the underlying resin chemistry when anticipating provisional restoration discoloration and advising patients on dietary habits. The duration of intraoral service significantly influences the degree of discoloration and surface roughness of provisional restorations. The longer a provisional restoration remains in the oral environment, the greater its susceptibility to color changes and surface degradation. This deterioration is largely affected by diet, oral hygiene practices, chemical reactivity, and water sorption [39].

As noted in Table 3, the conventional PMMA powder/liquid system showed the greatest color change, since the conventional PMMA powder/liquid system relies on benzoyl peroxide–amine redox initiation without added cross-linkers. This yields a network with a lower degree of conversion and substantial unreacted methyl methacrylate monomer. The high residual monomer and linear PMMA chains create microvoids and a more polar polymer matrix that readily absorbs water and hydrophilic chromogens (e.g., coffee pigments), driving larger shifts in lightness (L*) and chromatic axes (a*, b*) [40,41]. On the other hand, the multifunctional methacrylate formulation of the 3D printing and milled provisional resin uses multifunctional monomers, yielding a higher cross-link density than conventional PMMA, reducing overall water uptake [15].

### Clinical Implications and Limitations

The present findings have significant clinical implications, particularly in selecting provisional crown materials for patients requiring long-term provisionalization. Three-dimensionally printed and bis-acryl provisionals are superior due to their enhanced marginal adaptation and color stability, reducing the risk of microleakage, bacterial infiltration, and esthetic concerns. However, this study has certain limitations. The in vitro nature of the experiment does not account for the dynamic intraoral environment, which includes masticatory forces, pH fluctuations, and salivary enzymes, all of which can affect the longevity and performance of provisional restorations. Future in vivo studies should be conducted to validate these findings under clinical conditions.

## 5. Conclusions

Within the limitations of this study—which used multiple materials and fabrication techniques—the 3D-printed restorations demonstrated the most favorable outcomes, including superior marginal integrity and color stability, followed by the bis-acryl restorations when compared with the acrylic and milled CAD/CAM restorations. However, using different compositions and fabrication methods in a single experiment introduces confounding factors that can limit direct clinical recommendations. Future research should focus on more controlled designs that isolate either the material composition or fabrication technique to provide more explicit clinical guidelines. In addition, long-term in vivo studies, mechanical fatigue testing, and patient-reported outcomes are essential for a comprehensive understanding of provisional restorations’ durability and esthetic performance.

## Figures and Tables

**Table 1:** Composition of all examined temporary crown materials.

Product	Category	Composition	Manufacturer
VeriModel™OS	3D printing	Methacrylate oligomers and monomers, acrylate oligomers and monomers, and phosphine oxide	WhipMix, Louisville, KY, USA
SNAP^®^	Acrylic self-cure resin	Auto-polymerizing, powder/liquid, ethyl methacrylate resin, butylated hydroxytoluene (BHT)	Parkell, Edgewood, NY, USA
MaxiTemp HP	Bis-acryl self-cure resin	Methacrylate monomers, barium glass, silica, bis-acrylics	Henry Schein Inc., Melville, NY, USA
Telio	CAD/CAM	Cross-linked polymethyl methacrylate (PMMA)	Ivolar Vivadent, Ellwangen, Germany

**Table 2:** Data for all four groups and averages.

Groups	Count	Sum	Average	Variance
3D printing	19	2.218	0.11674	0.00916
Acrylic	20	6.022	0.30110	0.03046
Bis-acryl	20	2.349	0.11745	0.00762
CAD/CAM	20	3.511	0.17555	0.01135

**Table 3:** ANOVA of marginal integrity between four groups.

ANOVA	*SS*	*Df*	*MS*	*F*	*p-Value*
Between Groups	0.44781	3	0.14927	10.13891	0.00001
Within Groups	1.10419	75	0.01472		
Total	1.55199	78			

**Table 4:** Mean (±SD) CIELAB color change parameters after thermocycling.

Provisional Material	ΔE* (Total)	ΔL* (Lightness)	Δa* (Red–Green)	Δb* (Yellow–Blue)
3D-printed	0.46 ± 0.11	−0.18 ± 0.07	+0.12 ± 0.05	+0.31 ± 0.09
Bis-acryl	1.72 ± 0.32	−0.98 ± 0.18	+0.64 ± 0.13	+1.27 ± 0.29
Conventional acrylic	3.98 ± 0.47	−2.31 ± 0.35	+1.13 ± 0.21	+2.41 ± 0.44
Milled PMMA	0.89 ± 0.18	−0.42 ± 0.09	+0.21 ± 0.08	+0.69 ± 0.15

## Data Availability

All data has been included in this study.

## References

[1] Al-Humood H., Alfaraj A., Yang C.C., Levon J., Chu T.G., Lin W.S. (2023). Marginal Fit, Mechanical Properties, and Esthetic Outcomes of CAD/CAM Interim Fixed Dental Prostheses (FDPs): A Systematic Review. Materials.

[2] Angwarawong T., Reeponmaha T., Angwaravong O. (2020). Influence of thermomechanical aging on marginal gap of CAD-CAM and conventional interim restorations. J. Prosthet. Dent..

[3] Yao Q., Morton D., Eckert G.J., Lin W.S. (2021). The effect of surface treatments on the color stability of CAD-CAM interim fixed dental prostheses. J. Prosthet. Dent..

[4] Khng K.Y.K., Ettinger R.L., Armstrong S.R., Lindquist T., Gratton D.G., Qian F. (2016). In vitro evaluation of the marginal integrity of CAD/CAM interim crowns. J. Prosthet. Dent..

[5] Park J.Y., Lee J.J., Bae S.Y., Kim J.H., Kim W.C. (2016). In vitro assessment of the marginal and internal fits of interim implant restorations fabricated with different methods. J. Prosthet. Dent..

[6] Givens E.J., Neiva G., Yaman P., Dennison J.B. (2008). Marginal adaptation and color stability of four provisional materials. J. Prosthet. Dent..

[7] Mainjot A.K., Dupont N.M., Oudkerk J.C., Dewael T.Y., Sadoun M.J. (2016). From artisanal to CAD-CAM blocks: State of the art of indirect composites. J. Dent. Res..

[8] Rayyan M.M., Aboushelib M., Sayed N.M., Ibrahim A., Jimbo R. (2015). Comparison of interim restorations fabricated by CAD/CAM with those fabricated manually. J. Prosthet. Dent..

[9] Abdullah A.O., Tsitrou E.A., Pollington S. (2016). Comparative in vitro evaluation of CAD/CAM vs. conventional provisional crowns. J. Appl. Oral Sci..

[10] Alharbi N., Alharbi S., Cuijpers V.M., Osman R.B., Wismeijer D. (2018). Three-dimensional evaluation of marginal and internal fit of 3D-printed interim restorations fabricated on different finish line designs. J. Prosthodont. Res..

[11] Young H.M., Smith C.T., Morton D. (2001). Comparative in vitro evaluation of two provisional restorative materials. J. Prosthet. Dent..

[12] Davidowitz G., Kotick P.G. (2011). The Use of CAD/CAM in Dentistry. Dent. Clin. North Am..

[13] Van Noort R. (2012). The future of dental devices is digital. Dent. Mater..

[14] Al Wadei M.H.D., Sayed M.E., Jain S., Aggarwal A., Alqarni H., Gupta S.G., Alqahtani S.M., Alahmari N.M., Alshehri A.H., Jain M. (2022). Marginal Adaptation and Internal Fit of 3D-Printed Provisional Crowns and Fixed Dental Prosthesis Resins Compared to CAD/CAM-Milled and Conventional Provisional Resins: A Systematic Review and Meta-Analysis. Coatings.

[15] Jain S., Sayed M.E., Shetty M., Alqahtani S.M., Al Wadei M.H.D., Gupta S.G., Othman A.A.A., Alshehri A.H., Alqarni H., Mobarki A.H. (2022). Physical and Mechanical Properties of 3D-Printed Provisional Crowns and Fixed Dental Prosthesis Resins Compared to CAD/CAM Milled and Conventional Provisional Resins: A Systematic Review and Meta-Analysis. Polymers.

[16] Beuer F., Schweiger J., Edelhoff D. (2008). Digital dentistry: An overview of recent developments for CAD/CAM generated restorations. Br. Dent. J..

[17] Tahayeri A., Morgan M., Fugolin A.P., Bompolaki D., Athirasala A., Pfeifer C.S., Bertassoni L.E. (2018). 3D printed versus conventionally cured provisional crown and bridge dental materials. Dent. Mater..

[18] Ireland M.F., Dixon D.L., Breeding L.C., Ramp M.H. (1998). In vitro mechanical property comparison of four resins used for fabrication of provisional fixed restorations. J. Prosthet. Dent..

[19] Peng C.C., Chung K.H., Ramos V. (2020). Assessment of the Adaptation of Interim Crowns using Different Measurement Techniques. J. Prosthodont..

[20] Schwantz J.K., Oliveira-Ogliari A., Meereis C.T., Leal F.B., Ogliari F.A., Moraes R.R. (2017). Characterization of Bis-Acryl composite resins for provisional restorations. Braz. Dent. J..

[21] Peng C.C., Chung K.H., Yau H.T., Ramos V. (2020). Assessment of the internal fit and marginal integrity of interim crowns made by different manufacturing methods. J. Prosthet. Dent..

[22] Sakrana A.A. (2013). In vitro evaluation of the marginal and internal discrepancies of different esthetic restorations. J. Appl. Oral Sci..

[23] Bral M. (1989). Periodontal Considerations for Provisional Restorations. Dent. Clin. North Am..

[24] Doray P.G., Li D., Powers J.M. (2001). “Color stability of provisional restorative materials after accelerated aging”. J. Prosthodont..

[25] Gale M.S., Darvell B.W. (1999). Thermal cycling procedures for laboratory testing of dental restorations. J. Dent..

[26] McLean J.W., von Fraunhofer J.A. (1971). The estimation of cement film thickness by an in vivo technique. Br. Dent. J..

[27] Wu J., Xie H., Sadr A., Chung K.H. (2021). Evaluation of internal fit and marginal adaptation of provisional crowns fabricated with three different techniques. Sensors.

[28] Jalalian E., Younesi F., Golalipour S., Khorshidi S., Mahdavisaedabadi S.H., Sayyari M. (2023). Assessment of Marginal and Internal Adaptation in Provisional Crowns Utilizing Three Distinct Materials. J. Contemp. Dent. Pract..

[29] Cenci M.S., Pereira-Cenci T., Donassollo T.A., Sommer L., Strapasson A., Demarco F.F. (2008). Influence of thermal stress on marginal integrity of restorative materials. J. Appl. Oral Sci..

[30] Lopez D., Ziada H., Abubakr N.H. (2024). Influence of thermal aging on the marginal integrity of computer aided design/computer aided manufacturing fabricated crowns. J. Dent. Sci..

[31] Bayindir F., Kürklü D., Yanikoğlu N.D. (2012). The effect of staining solutions on the color stability of provisional prosthodontic materials. J. Dent..

[32] Aburaisi S., Basha A., Al Najjar K., Al Saqat H., Al Askar F., Al Nazer F. (2021). The colour stability of crystallized acetyl resin material in comparison to other restorative materials. An in-vitro study. BDJ Open.

[33] Song S.Y., Shin Y.H., Lee J.Y., Shin S.W. (2020). Color stability of provisional restorative materials with different fabrication methods. J. Adv. Prosthodont..

[34] Köroğlu A., Sahin O., Dede D., Yilmaz B. (2016). Effect of different surface treatment methods on the surface roughness and color stability of interim prosthodontic materials. J. Prosthet. Dent..

[35] Haralur S.B., Albarqi A.T., Alamodi A.G., Alamri A.A., Aldail S.A., Al-Qarni M.A., AlQahtani S.M., Alqahtani N.M. (2024). Comparison of Various Surface Treatment Procedures on the Roughness and Susceptibility to Staining of Provisional Prosthodontic Materials. J. Funct. Biomater..

[36] Haselton D.R., Diaz-Arnold A.M., Dawson D.V. (2005). Color stability of provisional crown and fixed partial denture resins. J. Prosthet. Dent..

[37] Radwan H., Elnaggar G., El Deen I.S. (2021). Surface roughness and color stability of 3D printed temporary crown material in different oral media (In vitro study). Int. J. Appl. Dent. Sci..

[38] Lopes-Rocha L., Mendes J.M., Garcez J., Sá A.G., Pinho T., Souza J.C., Torres O. (2021). The effect of different dietary and therapeutic solutions on the color stability of resin-matrix composites used in dentistry: An in vitro study. Materials.

[39] Elagra M.I., Rayyan M.R., Alhomaidhi M.M., Alanaziy A.A., Alnefaie M.O. (2017). Color stability and marginal integrity of interim crowns: An in vitro study. Eur. J. Dent..

[40] Alla R., Raghavendra K.N., Vyas R., Konakanchi A. (2015). Conventional and contemporary polymers for the fabrication of denture prosthesis: Part I–overview, composition and properties. Int. J. Appl. Dent. Sci..

[41] Zafar M.S. (2020). Prosthodontic applications of polymethyl methacrylate (PMMA): An update. Polymers.

